# Formulation, Delivery and Stability of Bone Morphogenetic Proteins for Effective Bone Regeneration

**DOI:** 10.1007/s11095-017-2147-x

**Published:** 2017-03-24

**Authors:** Inas El Bialy, Wim Jiskoot, M. Reza Nejadnik

**Affiliations:** Division of Drug Delivery Technology, Cluster BioTherapeutics, Leiden Academic Centre for Drug Research (LACDR), Leiden University, 2333 CC Leiden, The Netherlands

**Keywords:** Bone morphogenetic proteins, Growth factor, Protein formulation, Protein stability, Regenerative medicine

## Abstract

Bone morphogenetic proteins (BMPs) are responsible for bone formation during embryogenesis and bone regeneration and remodeling. The osteoinductive action of BMPs, especially BMP-2 and BMP-7, has led to their use in a range of insurmountable treatments where intervention is required for effective bone regeneration. Introduction of BMP products to the market, however, was not without reports of multiple complications and side effects. Aiming for optimization of the therapeutic efficacy and safety, efforts have been focused on improving the delivery of BMPs to lower the administered dose, localize the protein, and prolong its retention time at the site of action. A major challenge with these efforts is that the protein stability should be maintained. With this review we attempt to shed light on how the stability of BMPs can be affected in the formulation and delivery processes. We first provide a short overview of the current standing of the complications experienced with BMP products. We then discuss the different delivery parameters studied in association with BMPs, and their influence on the efficacy and safety of BMP treatments. In particular, the literature addressing the stability of BMPs and their possible interactions with components of the delivery system as well as their sensitivity to conditions of the formulation process is reviewed. In summary, recent developments in the fields of bioengineering and biopharmaceuticals suggest that a good understanding of the relationship between the formulation/delivery conditions and the stability of growth factors such as BMPs is a prerequisite for a safe and effective treatment.

## Use of Bone Morphogenetic Proteins for Bone Regeneration

### Introduction

Bone tissue has a unique self-remodeling and regeneration capability. Therefore, the standard treatment for bone defects such as fractures is composed of reduction and fixation of the fracture, acting as secondary aid to the self-healing process. In some instances (e.g., nonunion fractures of critical size defects, spinal fusions, open tibial fractures, and bone augmentation in dental implantology), the bone self-regeneration capacity is not sufficient and a more profound medical/surgical intervention to induce the formation of new bone is required. For such cases the use of autologous bone grafting, specifically iliac crest bone grafting (ICBG), has been considered as the “gold standard” treatment, as it provides a structural lattice that allows for cell migration, proliferation and tissue regeneration by employment of growth factors and osteoprogenitor cells [1]. This treatment, however, comes with multiple disadvantages, such as donor site morbidity represented in high postoperative pain, extended operating time with increased intra-operative blood loss, risk of infection and injury to nerves and blood vessels, possible postoperative gait disturbances, and limited availability of the graft especially in elderly patients [2, 3]. These limitations have driven the research towards tissue engineering approaches using bioactive molecules and materials.

### Nature of BMPs and their Applications

Bone morphogenetic proteins (BMPs) are naturally occurring molecules that were first identified by *Urist* in 1965 as proteins present in demineralized bone matrix that are capable of osteoinduction in ectopic sites in rats [4, 5]. Apart from BMP-1 (a metalloprotease), BMPs constitute a sub-class of the transforming growth factor β (TGF-β) superfamily [6]. To this date, around 20 BM Ps have been discovered; however, not all of them are in fact osteogenic molecules [7, 8]. BMP-2 and BMP-7 are, arguably, the strongest inducers of bone and cartilage formation. While BMP-4, BMP-5, BMP-6, BMP-8, BMP-9, and BMP-10 contribute to bone formation as well, BMP-3 and BMP-13 act as BMP inhibitors [9, 10]. The other BMP members are involved in developmental activities other than osteogenesis [8, 11].

A big share of the research efforts has been focused on the development of BMP-2 and BMP-7 drug products. After initial work using bovine BMPs, in the late 1980s, the molecular cloning of the human BMP genes was successfully achieved [12]. Since then, several BMP family members have been separated and in addition human recombinant BMP-2 and BMP-7 (further referred to as rhBMP-2 and rhBMP-7, respectively) were produced and purified for therapeutic applications [13, 14]. Evidence of their ability to induce bone in spinal fusions and nonunions in animal models led to their investigation in human clinical trials and the introduction of products to the biopharmaceutical market as a therapeutic replacement for ICBG.

### Structure and Properties of BMPs

A common denominator among BMPs is the presence of a cysteine knot involving 6 cysteine residues as well as a heparin-binding site [15]. These sites essentially interact with the endogenous macromolecules heparin/heparin sulfate present on cell surfaces and the extracellular matrix, resulting in the regulation of the bone formation process [16, 17]. Like all the other BMPs, BMP-2 and BMP-7 exist as homodimers where two BMP molecules are held together by a disulfide bridge through a 7th cysteine residue in their structure [18, 19]. This dimeric nature of BMPs is a necessary requirement for their biological activity, as the breakage of the disulfide bridges holding the molecules together renders the proteins inactive.

**Fig. 1:**
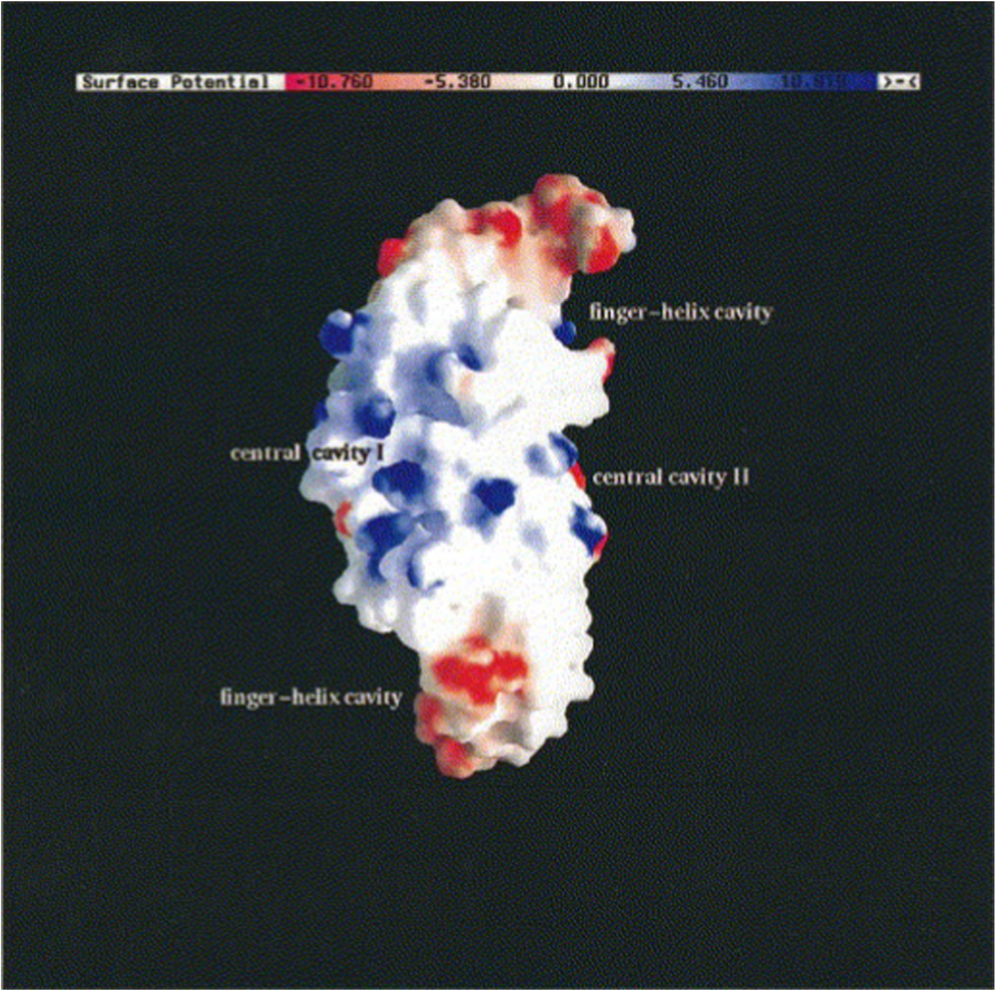
Surface charge density of rhBMP-2. Red and blue colors indicate negative and positive electrostatic potential, respectively. White color represents hydrophobic regions [18].

Human BMP-2 contains 114 amino acid residues and has a molecular weight of ~32 kDa [20]. BMP-7 consists of 139 amino acids and has a molecular weight of ~36 kDa [21, 22]. The molecular weight in both cases represents the dimeric existence of the molecules. All BMPs are basic proteins where they have their isoelectric points (pI) between 7.7 and 9, with BMP-2 and BMP-7 having very similar pIs at 8.2 [23] and 8.1 [21], respectively. Furthermore, they have abundant hydrophobic patches on their surface, represented in white in Fig. 1. Therefore, they show limited solubility at physiological pH, a property that is thought to be relevant to their pharmacological activity [18]. Rapid clearance is another feature of BMPs. For instance, when administered in buffer only, BMP-2 has a half-life time of ~7 min in non-human primates [24, 25].

An important parameter to consider when constructing a BMP product is that BMPs are pleiotropic proteins, meaning that they influence at least one or more molecular pathways beside their role in bone regeneration [26, 27]. Therefore, their diffusion to nearby tissues can result in unwanted ectopic bone formation, native bone resorption, and/or swelling of soft tissue [28]. These facts emphasize the importance of the incorporation of BMPs into delivery systems with programmable spatiotemporal release that would allow the presence of physiological doses of BMP only in the confined space which is limited to the defect region.

### Current BMP Products and Overview of Historical Events

Presently, there is one rhBMP-2 product on the market which is marketed as the INFUSE® Bone Graft Kit (Medtronic) in the US and as InductOS® Kit (Wyeth) in Europe. It is a lyophilized product containing rhBMP-2 at a concentration of 1.5 mg/ml after reconstitution, along with an absorbable collagen sponge (ACS) as a carrier for the protein. The product is commercially available at the total doses of 6 and 12 mg. Since the collagen sponge does not provide adequate mechanical support, the product needs to be combined with a supportive structure such as the LT-CAGE®, also produced by Medtronic. It is a titanium tapered cage that is implanted during the surgery as an interbody fusion device for spinal fusion procedures. The product has been introduced as an alternative treatment for bone grafting for multiple clinical conditions including spinal fusions, internal fixation of fractures, treatment of bone defects and reconstruction of maxillofacial conditions [29].

During the late 1990s and early 2000s, clinical trials were performed to compare the rhBMP-2/ACS treatment against the standard ICBG in anterior lumbar interbody fusion (ALIF) procedures. Results of these trials showed higher fusion rates for the rhBMP-2/ACS treated groups, and either similar or improved back and leg pain indices [30–32]. The impressive reported outcomes of the clinical studies resulted in the US Food and Drug Administration (FDA) approval in 2002 of the INFUSE® Bone Graft for spine fusion procedures employing the ALIF technique. Additional FDA approvals followed in 2004 for the use of rhBMP-2 to treat acute and open fractures of the tibial shaft, and in 2007 for oral maxillofacial applications [29]. The FDA approval, in turn, led to a marked increase in the use of rhBMP-2 in spinal fusion procedures from 0.69% of all fusions in the US in 2002 to 24.89% in 2006 [33, 34].

With this increased use of rhBMP-2 in the different orthopedic procedures, reports started to emerge regarding a series of safety concerns and possible side effects that were not published in the early clinical trials [35]. Ectopic/heterotopic bone formation [36, 37], dysphagia in cervical spinal fusions [38], vertebral bone resorption (osteolysis) [33, 39], postoperative radiculitis [40, 41], postoperative nerve root compression [42, 43], graft subsidence, and cage migration [33, 44] were among the frequently reported side effects. Mixed accounts were reported of the effect of rhBMP-2 on the incidence of retrograde ejaculation [45–48], and on its carcinogenic effects [49–54].

An extensive review by Carragee *et al.* [48] reassessed the efficacy and safety of the rhBMP-2 treatments published in 13 different clinical studies [30–32, 55–63]. The authors of the review stated that the thirteen clinical trial publications had consistently exaggerated the morbidity of the ICBG harvesting procedure and at the same time underestimated the side effects associated with the use of rhBMP-2, leading to false, or at least inflated, estimations of the reported rhBMP-2 safety and efficacy when compared with ICBG. After a revised assessment of the side effects associated with the use of rhBMP-2, which was reported to have “perfect” safety in the original studies, the authors concluded that the true risk to the patients is 10 to 50 times higher than that originally reported. For further investigation of such serious findings, the Yale University Open Data Access project team conducted a meta-analysis of individual-participant data [64]. The re-analyzed results considered the body of evidence strong enough for the initially reported effectiveness of the rhBMP-2 treatment but echoed the concerns related to the safety of the rhBMP-2.

While much of the effort has been focused on rhBMP-2 development and assessment, BMP-7 also had a share of research aiming at its introduction as a commercial product to the biopharmaceutical market. The results of the first clinical trial for rhBMP-7 in cases of tibial nonunions showed no significant difference between the rhBMP-7 treated group and the ICBG treated group in terms of safety and efficacy; moreover, they failed to prove superiority of the rhBMP-7 treatment over the autogenous bone graft [65]. In 2001 and following this trial, rhBMP-7 received a limited FDA approval in the US under a Humanitarian Device Exemption for treatment of recalcitrant tibial nonunions, and was subsequently introduced to the market as OP-1 by Stryker Corporation in the US and as Osigraft in Europe. The OP-1 product is a putty containing 3.5 mg of rhBMP-7, 1 g of type I bovine collagen matrix, and 230 mg of the putty additive carboxymethylcellulose sodium (CMC) to be reconstituted using sterile saline [66, 67].

Consecutive trials studied the use of OP-1 in patients suffering from grade I or II spondylolisthesis. Whether OP-1 was administered as an adjunct to or as a replacement for ICBG, it was found to have similar results as the use of the autograft alone in terms of bone bridging and showed no significant side effects. Again, no statistically significant differences could be established between the two treatments [67–69]. A large-prospective-randomized-controlled-multicenter clinical trial was started in an attempt to obtain an FDA Premarket Approval (PMA), which allows for unlimited product usage as long as it meets the approved use [70]. The trial aimed to demonstrate non-inferiority of OP-1 against ICBG in treatment of patients with spondylolisthesis. However, it did not succeed in showing that OP-1 treatment is truly non-inferior to ICBG. As a consequence, in March 2009, an FDA advisory committee voted against the PMA request for OP-1. In 2010, Stryker Biotech sold the OP-1 assets to Olympus Biotech Corp., which later in 2014 discontinued the sale of its products, including OP-1, in the US. Therefore, there are currently no rhBMP-7 products on the market.

### Dosing of BMPs

It has been suggested that the high doses of administered BMPs are one of the main reasons behind the reported adverse events accompanying their use in bone repair procedures [44]. In all the previously discussed clinical trials and all surgical treatments involving INFUSE® bone graft kit, rhBMP-2 has been delivered at a supraphysiological concentration [24, 44, 71–75]. Typically, the exogenous therapeutic rhBMP-2 is administered at a dose in the milligram range, which exceeds one million times the physiological protein amount, produced in nanograms under normal bone repair conditions [72]. The supraphysiological BMP-2 doses administered locally during the surgical procedure in clinical studies have been connected with complications, such as generalized hematomas in soft tissue [76], exaggerated inflammatory response in proximal humeral fractures [77], unicameral bone cysts [78], and infections in open tibial fractures [73, 79].

Furthermore, in an attempt to introduce a new rhBMP-2 product to the market, an Investigational Device Exemption study was conducted using a high dose (40 mg) rhBMP-2 product (called AMPLIFY, by Medtronic) on patients with single-level degenerative lumbar disease [80]. After the two-year follow up of the trial, the outcomes reported the incidence of eight cancer cases in the patients treated with AMPLIFY as opposed to two cancer cases in the control group receiving ICBG treatment [58, 71]. In 2013, the FDA denied AMPLIFY a pre-marketing approval following the occurrence of additional cancers in the AMPLIFY treated group [71]. It is noteworthy that INFUSE uses 6 and 12 mg doses and the product has not been reported to enhance the risk of cancer significantly [64].

On the other hand, BMPs show dose-dependent efficacy, where lower doses were inferior with regard to amount, quality, and time required for bone formation when tested in spinal fusion procedures in non-human primates [81, 82]. Similarly, in a study in human patients with open tibial fractures, an rhBMP-2 dose of 6 mg showed 44% increase in cases of nonunions requiring secondary interventions compared to a dose of 12 mg [83]. The reduced efficacy associated with lower BMP doses and compromised safety of the higher doses form a dilemma for acquiring an optimal dose regimen. This has stimulated the search for improved delivery systems that allow for sustained and controlled release of the BMP.

### Aim of this Review

This review addresses the carrier properties (e.g., material and configuration) and in particular the stability of BMP molecules in the formulations, as these are all parameters that affect the therapy outcomes of BMPs [23]. It has to be realized that a number of concerns regarding the efficacy and safety of the BMP-based approaches for bone formation have been reported in the past decade. At the same time, it has been shown that the efficacy and some of the reported side effects of the BMPs can be controlled by improving their delivery [84]. The raise of these concerns have, time-wise, coincided with exploding scientific discoveries in the field of protein pharmaceuticals and in particular concerning our understanding of how instability of proteins can lead to loss of efficacy and increased immunogenicity [85–87]. Therefore, some of the reported side effects and challenges arising from the utilization of BMP-based therapies could be related to the protein stability issues, as discussed in this review. Clearly, any potential hint from the literature could lead to game-changing solutions towards safer and more effective BMP-based therapies.

## BMP Delivery Systems

The delivery system can be considered as the most important parameter regarding the delivery of BMPs. A properly designed delivery system is administered locally via surgery and would be able to localize the BMP only at the target repair site. Such a delivery construct would have a built-in release system that is able to keep the local BMP concentration over time high enough to induce osteoinduction and the systemic concentration low enough to avoid the adverse events encountered with supraphysiological doses of the BMP [88]. Superiority in terms of bone regeneration and newly-formed bone quality was demonstrated in a rat model with femoral defects when controlled spatiotemporal BMP release from a hybrid system composed of alginate hydrogel contained in a nanofiber mesh was compared with the commonly used absorbable collagen sponge [89].

Furthermore, bone formation by using relatively low dose rhBMP-2 (8 μg/ml) was achieved in mice with critical size calvarial defects using a semi-synthetic PEGylated fibrinogen delivery system. Upon subcutaneous implantation, the hydrogel acts as a matrix that can regulate the release of rhBMP-2 in physiological doses at its implantation site [90]. Another delivery system was constructed by allowing supramolecular nanofibers to form gel networks within the pores of ACS. These nanofibers have an affinity for binding BMP-2 with the help of heparin sulfate, and thus increase the retention time at the site of administration/implantation and allow for lower doses. With this delivery system, bone regeneration was achieved in a rat critical-size femoral defect model using BMP-2 doses (1 μg) that were one order of magnitude lower than the previously reported model’s minimum threshold for healing (11 μg) [73]. These and other studies suggest that the *in vivo* spatiotemporal release kinetics of BMP in a delivery system will be affected by the choice of carrier material, the protein incorporation method and the type of protein-carrier interaction, as well as by the carrier’s physical configuration [91].

For bone regeneration applications, tissue engineering growth factors in general need to be delivered with a scaffold for the purposes of providing mechanical support as well as a three-dimensional (3D) matrix that allows for the release of the payload and growth of the new tissue. Metal scaffolds made of titanium are commonly used support scaffolds for bone repair applications [92] as they can be processed into macroscopic fiber meshes and porous scaffolds, and thus create a suitable environment for tissue growth and allow for its integration with the native bone [93, 94]. However, rhBMP molecules incorporated into titanium support scaffolds are either adsorbed to the surfaces or are superficially entrapped and therefore can be rapidly released *in vivo* [95]. The incorporation of one or more protein carriers is thus essential for sustaining the rhBMP release *in vivo.*

**Fig. 2:**
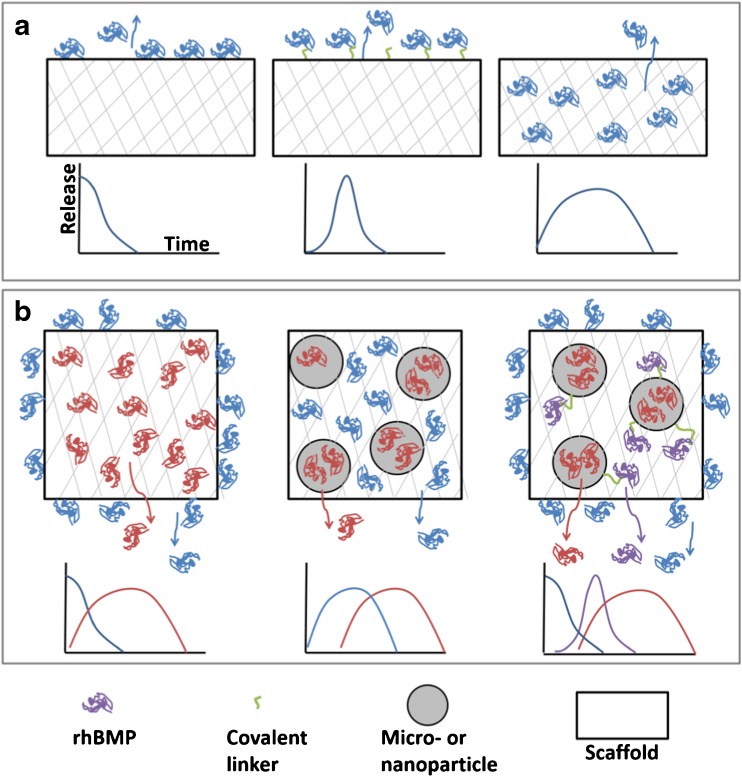
Illustrative diagram of BMP immobilization approaches. (**a**) rhBMP immobilization methods on single-material scaffolds: adsorption (left), chemical immobilization (middle), and physical entrapment (right). A postulated release profile is displayed beneath each method. (**b**) Examples of potential rhBMP multiple immobilization methods on either single-material or composite scaffolds: combination of adsorbed and physically immobilized BMP (left), particle-encapsulated BMP incorporated into a scaffold along with BMP directly physically immobilized into the scaffold (middle), and additional chemical immobilization of the BMP onto the composite scaffold (right). A postulated release profile is shown below each method.

The carrier material can be either formulated into a scaffold that serves as both the required mechanical support and the delivery system for rhBMPs, or formulated only as the delivery system which is then incorporated into/onto a separate scaffold. Examples of the latter include the formulation of rhBMP-2 into polymeric carriers such as hyaluronic acid (HA) [96] and polylactic acid [97] which were then coated onto the surface of titanium scaffolds and tested in rats and sheep, respectively. The formulation of the delivery system into different configurations (e.g., solid or hydrogel scaffolds, micro- and nanoparticles) and the method of the protein incorporation/immobilization with the carrier are all factors that influence the overall conditions of delivery and, consequently, the clinical effects. Figure 2 demonstrates the different carrier configurations and BMP immobilizations strategies.

### Carrier Materials

Different types of carrier materials have been investigated for their capability of delivering rhBMPs and assessed for their general performance in achieving osteoinduction. Different carrier materials have been commonly classified according to their nature of origin and chemical composition into four main classes: natural polymers, synthetic polymers, inorganic materials, and their composites. Each class has advantages and disadvantages over the others. This is why no carrier for the delivery of BMPs is considered universally accepted, but rather some carriers become more suitable than others with respect to a certain application. This section contains an overview of the most commonly researched members of each class, their general advantages and drawbacks, and examples of the findings regarding their use in delivery of rhBMPs (see Table I for an overview of the carriers covered in this review). For a more extensive review of all the available carrier materials studied in combination with rhBMPs, the reader is referred to other detailed review articles [24, 84, 98, 99].

**Table I:** Overview of the Carriers Covered in this Review

Class	Types	Delivery form(s)	Preclinical studies	Reference
Natural polymers	Collagen	Powders	BMP-2 in:	
		Membrane films	- Maxillofacial reconstruction in Rhesus monkeys	[104]
		Aqueous forms	- Rabbit ulna osteotomy model	[103]
		Gels	- Healing in goat tibial fracture model	[102]
		Nanofibers	BMP-7 in:	
		Putty	- Healing of segmental defects in non-human primates	[101]
		Absorbable sponge	- Lumbar vertebral interbody fusion in sheep	[100]
	Hyaluronic acid/Hyaluronan		BMP-2 in:	
		Gels	- Dog alveolar ridge defects	[113]
		Scaffolds	- Mid-tibial unions in rabbits	[114]
		Aqueous forms	- Rat calvarial defects	[115]
	Gelatin		BMP-2 in:	
		Hydrogel	- Ulnar bone segmental defects in New Zealand White rabbits	[117]
		Microparticles on a composite scaffold	- Ectopic bone production in a mouse model	[118]
	Fibrin		BMP-2 in:	
		Hydrogel	- Calvarial bone defects in New Zealand White rabbits	[119]
	Chitosan		BMP-2 in:	
		Film	- C2C12 cell line of mouse muscle myoblast cells	[121]
	Alginate		BMP-2 in:	
		Aqueous form	- Posterolateral spine fusion in rabbit model	[122]
	Silk		BMP-2 in:	
		Film	- Cell culture inserts	[126]
		3-D porous scaffolds	- Critical sized cranial defects in mice	[125]
		Microparticles	- Rat ectopic model	[124]
Synthetic polymers	Poly-α-hydroxy acids		BMP-2 in:	
	i) Polylactic acid (PLA)	Aqueous form	- Canine posterolateral spinal fusion model	[128]
		Preshaped implants	- Mandibular bone repair in rats	[127]
	ii) Polyglycolic acid (PGA)		BMP-2 in:	
		Nonwoven fabric made from PGA fibers, scaffolds, nanoparticles	- Induction of bone is Wistar rat thigh muscle	[131]
			- Critical-sized calvarial defects in rats	[130]
	ii) poly(D,L-lactide-co-glycolide) (PLGA)		BMP-2 in:	
		Microparticles	- Intramuscular bone induction in mice	[136]
		Implants	- Mandibular defects in canine model	[135]
		3-D scaffolds	- Differentiation of rabbit bone marrow stromal cells	[134]
		Capsules	- Segmental bone defects in rabbit radius	[133]
			BMP-7 in:	
		Gels	- Bone formation from rabbit skeletal muscle cells	[132]
	Polyethylene glycol (PEG)		BMP-2 in:	
		Hydrogel	- Critical-sized defects in rat crania	[137]
	Poly-ε-caprolactone (PCL)		BMP-2 in:	
		3-D scaffolds	- Osteoinduction in bone marrow stromal cells	[140]
	Polypropylene fumarate (PPF)	Porous scaffolds	BMP-2 in:	[141]
			- Goat ectopic implantation model	
	Poloxamers	Freeze-dried powder	BMP-2 in:	[142]
			- Bone induction in Swiss-Webster mice	
	Block copolymers		BMP-2 in:	
	i) PLA-PEG	Pellets	- New bone induction in dorsal muscles of mice	[145–147]
	ii) PLA-DX-PEG		BMP-2 in:	
		implant	- New bone induction in dorsal muscles of mice	[148]
Inorganic materials (ceramics)	Calcium Phosphate Materials		BMP-2 in:	
	i) Hydroxyapatite	Fiber mesh	- Rat posterolateral spinal fusion	[153]
		Cement	- Rabbit unilateral radii defect	[154]
			BMP-7 in:	
		Porous scaffold	- Spinal fusion in sheep model	[155]
			- Orthotopic calvarial defects in baboons	[156]
	ii) β-tricalcium phosphate (TCP)		BMP-2 in:	
		Porous multi-cylinder scaffolds	- Long intercalated rib defects in dogs	[160]
		Cement	- Trepanation defects in sheep	[161]
		Granules	- Spinal fusion in canines	[162]
	iii) Biphasic calcium phosphate (BCP)		BMP-2 in:	
		Scaffolds	- Rat calvarial bone defects	[164]
			- Intertransverse spine arthrodesis in non-human primates	[82]
Composites	Semi-synthetic polymers			
	PEGylated fibrinogen	Hydrogel	BMP-2 in critical size calvarial defects in mice	[90]
	RGD-Alginate	Nanofiber mesh hydrogel	BMP-2 in bilateral critical size defects in rats	[166]
	PCL-Collagen	Nanofibrous scaffold	BMP-2 in in vitro activation of pre-osteoblasts	[167]
	CMC-Collagen	Putty	BMP-7 in critical size defects in ovine tibiae	[168]
	Polymers + Ceramics Collagen-Biphasic calcium phosphate	Scaffold	BMP-2 in rabbit calvarial defects	[173]
	Three-component PEG-PCL-PEG copolymer-collagen-n-hydroxyapatite	Hydrogel	BMP-2 in cranial defects in rabbits	[174]

*Natural polymers* have been largely investigated for the delivery of rhBMPs because of their favorable characteristics that include biocompatibility, biodegradability, and solubility in physiological environments. Since most natural polymers are derived from animals, they possess the disadvantages of immunogenicity and the potential risk of transmitting animal-originated pathogens as well as the general difficulty in their processing. Collagen has been the most extensively used carrier for delivery of rhBMPs, and it is the carrier employed in both commercial rhBMP products (INFUSE® and OP-1®). The facts that collagen is the most abundant non-mineral component of bones and that it can be easily isolated and purified enzymatically from various animal species make it a highly favorable carrier candidate for rhBMP. Collagen has been fabricated as powder, membrane films, aqueous forms, gels, nanofibers, and the most common, absorbable sponge [95, 99–104].

Despite its optimal biocompatibility, collagen possesses a number of disadvantages. As a scaffold, collagen is mechanically weak and therefore, when implanted in an environment where the sponge is compressed by surrounding muscles and tissue, undesirably high doses of rhBMPs could be locally released [24, 95]. Furthermore, the biodegradation of the collagen matrix is unpredictable and difficult to control, resulting in undefined release kinetics of the entrapped protein [105]. Even though the rhBMP-2 retention at the defect site was prolonged by its incorporation into a collagen sponge when compared to buffer only, it was shown *in vivo* that only 5% of the protein remains within the collagen after 2 weeks due to initial burst release [106, 107]. In addition, collagen possesses immunogenic properties due to its common extraction from bovine and porcine skin, where 20% of patients receiving rhBMP-2/ACS were found to have developed antibodies against type I collagen [99]. Another problem encountered with collagen is sterilization difficulty, where heat sterilization causes complete or partial denaturation where the collagen helices become irreversibly damaged [106, 108]. Thus, usually ethylene oxide is used to sterilize the collagen sponge. However, this method of sterilization poses the risk of affecting the rhBMP’s release kinetics and structural integrity, and consequently its bioactivity [109], as was demonstrated by the reduced bone-inducing capacity of the extracted BMP after exposure to ethylene oxide [110–112].

Hyaluronic acid (HA), also called hyaluronan, is another natural polymer which has been studied for delivery of rhBMPs. Successful bone regeneration was reported with the use of HA as a carrier for rhBMP-2 in dog alveolar ridge defects [113], mid-tibial non-unions in rabbits [114], and rat calvarial defects when surgically administered in combination with mesenchymal stem cells [115]. When compared with a composite carrier made of collagen, hydroxyapatite and tricalcium phosphate, HA based delivery of rhBMP-2 produced larger bone and osteoid volumes [116].

Gelatin, which is denatured collagen, is another promising carrier for rhBMP-2. rhBMP-2 formulated in a macroscopic gelatin hydrogel was shown to be capable of inducing osteoinduction in ulnar bone segemental defects in skeletally mature New Zealand White rabbits [117]. Similarly, rhBMP-loaded gelatin microparticles in a poly propylene fumarate scaffold showed controlled and sustained release in a mouse model [118].

Fibrin, derived from blood clots, has also been used as a carrier for rhBMP-2 and the construct has significantly increased the formation of bone in calvarial bone defects in New Zealand White rabbits [119]. Fibrin along with HA and type 1 collagen in combination with heparin and rhBMP-2 demonstrated complete bone healing in a cranial implant model [120]. Other natural polymers that were studied as carriers for rhBMP include chitosan (a cationic copolymer prepared from chitin) [121], alginate (a polysaccharide obtained from sea weed) [122], and silk [123–126].

Owing to their flexible and easily controlled design, *biodegradable synthetic polymers* have been investigated as carriers for rhBMPs in bone tissue engineering applications. Poly-α-hydroxy acids are commonly used *synthetic polymers* in growth factor delivery; and their capacity for delivering rhBMP has been investigated [24]. rhBMP-2 successfully induced bone formation in various animal models when delivered by a matrices of polylactic acid (PLA) [127, 128], polyglycolic acid (PGA) [129–131], and their copolymer poly(D,L-lactide-co-glycolide) (PLGA) [132–136]. Other synthetic polymers that have been studied in combination with BMPs include polyethylene glycol (PEG) [137], polyanhydrides [138, 139], poly-ε-caprolactone (PCL) [140], polypropylene fumarate (PPF) [141], and poloxamers [142]. General advantages of this group of materials include their biocompatibility, hydrolytic biodegradability, low immunogenicity risk and eliminated possibility of disease transmission in addition to their general ease of use, formability and design flexibility [98, 143, 144]. An additional advantage of these materials over natural polymers is their ability to tailor the mechanical strength, adhesiveness and degradability according to their clinical use requirements through manipulating the polymer structure [98]. An approach that is often followed is the synthesis of block copolymers by polymerizing chains of different blocks in an attempt to control/manipulate one or more of the polymeric delivery system’s characteristics, such as its release kinetics. An example is the incorporation of rhBMP-2 in a delivery system based on PLA-PEG copolymer that is implanted in the form of a viscous liquid or pellets [145–147]. Even though PLA-PEG proved to be useful as a matrix for osteoinductive rhBMP-2, it was found that its degradation was too slow and that some of the carrier material remained at the center of the formed ossicles. Keeping the polymer molecular weight constant, para-dioxanone molecules were randomly inserted into the PLA segments of the PLA-PEG polymer creating the polymer PLA-DX-PEG. This modification aided in optimizing the degradation kinetics of the polymer. Using it as a delivery system for rhBMP-2 resulted in complete replacement of the implants by new bone without detectable polymer remnants inside the formed ossicles [148]. Many of the synthetic polymers, however, have the disadvantage of acidic breakdown byproducts that lower the local pH and potentially increase the associated risk of excessive inflammatory responses. Moreover, this acidification as well as the hydrophobic character of polymers like PLGA may compromise the protein stability [149]. Retarded clearance rate, lack of biological function, and chronic inflammation associated with high molecular weight polymers are other drawbacks encountered with the use of some synthetic polymers [95, 98, 99].

*Inorganic materials* (mainly ceramics) are another class of carrier materials that are investigated for delivery of rhBMPs. Calcium phosphate materials are the most common inorganic materials used in bone tissue regeneration because of their established ability for osteoconduction [150–152]. According to their chemical composition, the most used calcium phosphates are subdivided into three main categories: hydroxyapatite, β-tricalcium phosphate, and a combination of both called biphasic calcium phosphate [24]. Administration of rhBMP incorporated into a hydroxyapatite carrier demonstrated bone formation with rhBMP-2 in rat posterolateral spinal fusion [153] and rabbit unilateral radii defect [154]. The same carrier has also been used for rhBMP-7 in spinal fusion in a sheep model [155] and in baboon orthotopic calvarial defects [156]. In contrast, rhBMP-2 delivered with hydroxyapatite failed to demonstrate any capability of bone formation after subcutaneous implantation in rats [157], which was later reasoned to be due to high affinity between the carrier material and the protein [99].

Disadvantages associated with hydroxyapatite are related to its brittleness, poor resorbability, and insufficient mechanical strength [24]. A comparison held by Tazaki *et al.* between hydroxyapatite and β-tricalcium phosphate revealed the superiority of the latter owing to its relatively slower rhBMP-2 release rate [158]. For this reason and in addition to its chemical similarity to the normal bone structure, β-tricalcium phosphate has been the most commonly used bone graft substitute [150]. Furthermore, its biocompatibility, degradability, and low immunological and toxic reactions make it a potentially promising carrier for BMPs in bone tissue engineering [95, 159]. rhBMP-2 delivered by β-tricalcium phosphate in the form of solid cylinders was able to repair long intercalated rib defects in dogs [160], fill trepanation defects in sheep [161], and achieve posterolateral lumbar interbody fusion in dogs [162].

Biphasic calcium phosphate has been investigated to employ the different resorbability characteristics of hydroxyapatite and β-tricalcium phosphate to control the degradation kinetics by varying between their ratios [163]. Biphasic calcium phosphate, formed of hydroxyapatite and β-tricalcium phosphate in different ratios, demonstrated enhanced bone formation in a rat calvarial defect model [164] and in non-human primate intertransverse process spine arthrodesis [82]. Phase separation in case of administration by injection, lack of intrinsic macroporosity to allow cell infiltration and low mechanical tensile and shear properties compared to bone and other materials are all among the main disadvantages of calcium phosphate materials [98]. However, some of the problems could be resolved through formulation modifications, such as increasing macroporosity by the addition of gas producing excipients to induce granulation or to form pores [165].

In recent years, the trend has shifted towards delivering rhBMP using *Composite* carriers of different origins instead of a single carrier material. Such an approach would allow the designer to combine the benefits of the multiple materials to optimize the properties and to overcome some of the encountered limitations. The fabrication of semi-synthetic polymers was introduced to combine the controlled release advantages of synthetic polymers with the biocompatibility of natural polymers. These semi-synthetic polymers were successful in delivering rhBMPs and promoting osteoinduction in many studies. Recent examples include the use of PEGylated fibrinogen with low dose rhBMP-2 in critical size calvarial defects in mice [90], RGD-alginate hydrogel containing rhBMP-2 in bilateral critically-sized femoral bone defects in rats [166], and poly-ε-caprolactone (PCL) combined with collagen and low dose rhBMP-2 in *in vitro* MC3T3-E1 cells (pre-osteoblasts) [167]. As for delivering rhBMP-7, a putty composed of a combination of carboxymethyl cellulose (CMC) and collagen was investigated for its effectiveness for osteoinduction in critical size defects in ovine tibiae [168], and in spinal fusion procedures in ovariectomized female (osteoporotic) rats [169].

Composites with the addition of polymers (natural or synthetic) to ceramics have been synthesized with the purpose of improving the handling, porosity, and in some cases injectability of the ceramic carriers [170–172]. As a recent example, a disk-shaped, solid composite of collagen and biphasic calcium phosphate was prepared for rhBMP-2 delivery in rabbit calvarial defects and showed superiority over collagen-free biphasic calcium phosphate in terms of decreased burst release and bone regeneration [173]. More complex three-component composites were also synthesized for the purpose of bone regeneration, as demonstrated by the achieved injectability and thermo-sensitivity of the novel hydrogel PEG-PCL-PEG copolymer/collagen/n-hydroxyapatite [174].

### Carrier Configurations and Protein Incorporation

The simplest forms of carrier configurations are micro- or nanoparticles acting as simple depot delivery systems for the BMPs without contribution to the mechanical support functions. Besides, these delivery systems are generally considered cheap, simple, and efficient vehicles for drug delivery and/or targeting [84]. An early study tested the delivery of PLA microparticles for delivery of BMPs for bone formation in rats [175]. However, PLGA has caught the focus owing to its relatively controllable biodegradability by changing its PLA and PGA ratios [84, 176], and has thus been more thoroughly investigated for the delivery of rhBMPs in the forms of particles with a wide range of sizes from 430-μm microparticles [177] down to 300-nm nanoparticles [178]. To maintain the particles at the defect site for the essential local release of the incorporated BMP, they need to be retained within a scaffold. PLGA microparticles containing rhBMP-2 have commonly been incorporated in calcium phosphate cement scaffolds, which further prolong the release, an effect which has been attributed to possible affinity between rhBMP-2 and the scaffold [178–180]. Wei *et al.* encapsulated rhBMP-7 in PLGA nanospheres that were incorporated into a PLA scaffold. It was concluded that the carrier was able to deliver rhBMP-7 in a time-controlled manner and was able to significantly induce bone formation in a rat model [181].

Natural polymers were used to create BMP-containing microparticles as well. Osteoinduction was promoted upon rhBMP-2 delivery via nanoparticles made of dextran in rabbit bone marrow stem cells, and via microparticles made of the composites chitosan-alginate and dextran-gelatin [182] in rabbit bone marrow stem cells and *in vivo* in canine defects, respectively. It is well established that the use of particulate delivery systems such as micro- and nanoparticles bears an immunogenicity risk, as they are readily taken up *in vivo* by dendritic cells and macrophages initiating an immune response against the delivered protein/peptide. This risk, when added to the immunogenic nature of BMPs [183], could be detrimental for the therapy. Therefore, it would be wise to investigate the effect of delivering rhBMPs via this type of delivery systems on the expression of antibodies against the protein [85, 184].

Carriers have also been fabricated into macroscopic hydrogels and porous solid scaffolds that may contribute to the required mechanical support for 3D cell growth beside their role in delivering BMPs. Examples of the use of natural polymer scaffolds include all the animal and clinical studies utilizing rhBMP-2 soaked into collagen sponge (ACS) scaffolds as well as rhBMP-2 formulated into porous HA scaffolds [185]. BMPs have also been delivered by using composite solid scaffolds, e.g., chitosan-PGA [186], gelatin-β-tricalcium phosphate [187], and PLA-PEG-calcium hydroxyapatite [188]. Hydrogel scaffolds offer another configuration for the carrier materials used for delivery of BMPs. Unlike solid scaffolds, hydrogels are fabricated to contain a large amount of water and are characterized by swelling through increasing the water content upon implantation *in vivo*. This highly hydrated state allows for the free diffusion of oxygen and nutrients into the scaffold, and thus provides an optimum environment for the new bone tissue ingrowth [189–193]. Hydrogels are synthesized by crosslinking the branches of hydrophilic polymers using a bridging agent; where the water content depends on the type and concentration of the molecules and the bridging agent [95]. Examples of incorporation of BMPs within hydrogel scaffolds include the inclusion of rhBMP-2 into gelatin hydrogels with different water contents [117, 194], and rhBMP-2 along with human mesenchymal stem cells incorporated into a HA hydrogel administered for rat calvarial defect regeneration [115]. Another type of scaffolds composed of 3D nanofiber structure prepared by using an electrospinning technique. This type of structure provides a high surface area-to-volume ratio, thus enabling the adhesion and proliferation of osteogenic cells. Electrospun scaffolds prepared from chitosan [195], silk [196], and PCL-PEG [197] are selected examples investigated for their ability to deliver rhBMP-2.

The release pattern of the bone morphogenetic protein depends greatly on the type of interaction between the protein molecules and the carrier and generally the way the molecules are incorporated into the delivery system (Fig. 2). Physical adsorption to the delivery system’s surface is considered the simplest form to deliver the protein where the prefabricated scaffold is dipped into the protein solution and left to dry, in which there is no specific affinity between the protein and carrier molecules. The main disadvantage of this interaction is that the adsorption and drying may result in alteration of the conformational structure of the protein molecules with the possibility of affecting its bioactivity [91]. Physical entrapment of the protein within the delivery system material is another way of protein incorporation. This technique usually takes place by mixing the BMPs with the carrier material in its liquid form followed by phase change, such as gelation, leading to entrapment of the protein molecules. In a slightly different format, usually utilized with natural polymer sponges and hydrogels, the carrier is soaked in the protein solution just prior to implantation (the method used in commercially available product INFUSE®), allowing the protein to be loaded into the pores of the carrier material. When such a delivery system is subjected to the *in vivo* physiological environment, however, the protein may be released in a rapid uncontrolled fashion by diffusion through the delivery system and/or by degradation of the carrier material.

Covalent coupling of BMPs to the carrier material is a way to circumvent the limitations of surface adsorption and physical entrapment techniques for a more stable and sustained release. The immobilization depends on the presence of essential functional groups both on the protein and carrier molecules that would allow for the formation of a suitable covalent bond through bifunctional crosslinking or derivatizing reagents. One major drawback associated with the covalent bonding is that the drug substance is chemically altered, which may result in alteration in activity and interaction of the molecule with its environment. Modification of the drug substance may also lead to complications with respect to regulatory approvals of the product. Additionally, concerns have been raised regarding the effect of chemical coupling on changes in proteins structure and bioactivity and safety in general [198], and BMP-2 in particular [199]. Furthermore, the covalent bonding restricts free diffusion of the protein molecules within its microenvironment which might hamper the interaction with the appropriate receptors for osteoinduction.

Encapsulation of BMPs into micro- and nanoparticles may bypass most of the aforementioned issues regarding rapid release, however, many of the encapsulation techniques involve harsh conditions, such as the use of organic solvents, exposure to interfaces and acidic environment, all of which may subject the protein molecules to physical and/or chemical instability resulting in diminished bioactivity among other complications [24, 200, 201].

## Stability of BMPs

Proteins existing in their native state usually express low stability. Even minor changes in their surrounding environments can account as stress for the proteins, which may lead to chemical changes (e.g., oxidation and deamidation), physical changes (e.g., unfolding or misfolding, aggregation and particle formation), and surface adsorption, processes which may mutually influence each other. For instance, surface adsorption can potentially lead to changes in a protein’s structural integrity or aggregation, and conformational changes may trigger chemical degradation reactions [202]. Protein degradation can easily occur during the different processes that the protein needs to undergo until it reaches the patient, e.g., during production, storage, and administration. During such processes, the protein in its surrounding environment is subjected to several stress factors, such as elevated temperature, undesirable solution pH, presence of co-solutes in the aqueous solution such as salts, preservatives and surfactants, and contact with handling tools and other components of the formulation and delivery systems [203].

Despite its importance in efficacy and safety of the treatment, the subject of rhBMP stability and the factors affecting it has been addressed in only a handful of the published studies. Even fewer publications discussed the impact of such protein stability/instability on the *in vivo* activity and adverse effects. BMPs, like proteins in general, adopt a unique 3D structure in their aqueous environment that is essential for their bioactivity. Any alteration to this native protein conformation can lead to partial or total inactivation of the protein. Chemical and physical instability may lead to reduced or diminished activity and adverse effects, such as immunogenicity [87, 204, 205]. The presence of particulates may also lead to several side effects, such as local phlebitis, pain, swelling, inflammation, granuloma, anaphylactic or allergic reactions [86].

Although antibodies against administered rhBMP-2 and rhBMP-7 [35, 62, 70] and accompanying immediate pain [41, 206] have been reported following rhBMP-2 treatments, the possible relationship between the potential presence of soluble aggregates and/or particulates in BMP formulations and the observed adverse effects have, to our knowledge, not been studied. Furthermore, stability studies have, in most cases, not been reported on the rhBMP-7 formulation to determine whether or not the resultant inadequate efficacy was due to decreased bioactivity through denatured protein [70]. Similarly important and greatly neglected is the potential effect of protein degradation on the efficacy of BMP proteins. For instance, destabilized disulfide bonds, which commonly occur in neutral and basic environments, may result in their breakage and thus disruption of the dimeric nature and inactivation of BMPs [207]. Despite the fact that several studies state that a large amount of BMP was needed to get a biological response, little has been done to investigate whether the administered protein is still in its native form and active. Clearly, denaturation and subsequent inactivation could at least partially explain the need for BMP amounts up to a million-fold higher than biological concentrations for a successful effect.

A few limited studies have been published about the effect of elevated temperature (one of the stress factors BMPs commonly encounter during their production and formulation) on BMP stability. Crude human BMP was extracted from bone that was subjected to 60°C for 10 h and compared to the protein extracted from non-treated bone by sodium dodecyl sulfate polyacrylamide gel electrophoresis. The electrophoretic bands were found to be identical for the BMP from both bone sources, suggesting that the primary structure of the protein was not altered. Similar bone formation was also observed after implantation of the differently treated BMPs into thigh muscle pouches of five mice [208]. In another study, the bone-inducing activity of rhBMP-2 samples was determined before and after heating at different temperatures (50, 70, 90, 100, and 120°C) for different time periods (15 min, 1, 2, 4, and 8 h). The *in vitro* testing was done by adding the rhBMP-2 samples to cell cultures of MC3T3-E1 cells and testing the alkaline phosphatase activity (an early biomarker of osteoblastic differentiation and its expression is induced by BMP in a dose-dependent manner [209]) after 48 h. The rhBMP-2 bone-inducing activity was also tested *in vivo* by implanting freeze-dried collagen disks containing the rhBMP-2 samples into mice back muscles and examining the new bone formation into the disks after three weeks using radiography. The results of this study suggested that the rhBMP-2 is resistant to incubation at 50 and 70°C for short periods, while degradation starts at higher temperatures and/or long periods where heating at 120°C completely inactivated the protein [210].

The activity of BMP-7 extracted from human femoral bone head was tested in another study after exposure to both high and low temperatures. The aim of the study was to investigate the resistance of BMP-7 in tumor-bearing bones against freezing, pasteurization, and autoclaving treatments applied during biomechanical reconstruction procedures after bone tumor resection. The BMP-7 was subjected to −196, −73, 60, and 100°C for different time periods (20, 30 min, 10, and 12 h). The treated samples were analyzed *in vitro* for their BMP-7 content by using enzyme-linked immunosorbent assay (ELISA). A bioassay was also performed using NIH3T3 mouse fibrous cells and immunoblotting analysis to detect the amount of phospho-Smad, which is an indicator of the BMP-7 activity. The results showed that the BMP-7 retains its activity after freezing (−196 and −73°C) and thawing, while it partially loses it upon incubation at elevated temperatures (60 and 100°C) [211].

There are some points to note concerning the studies on the thermal stability of BMPs. Firstly, two of the studies are relatively old (2001 and 2005, respectively) and used only a few methods to study the protein’s stability *in vitro.* This fact would certainly compromise the significance of these results to the accurate physical stability information required nowadays [85, 212, 213]. Secondly, the studies by Izawa *et al.* and Takata *et al.* have used BMPs that were extracted from human bone rather than recombinant proteins, which again compromises the relevance of their results to the behavior of the marketed recombinant proteins to stress. Furthermore, these studies were focused on BMP activity and bone formation and did not study the actual effects of the temperature on the native structure and aggregation of the protein and therewith potential signs of adverse effects and toxicity/immunogenicity.

The pH of the solution environment is another important parameter that can greatly affect the rhBMP stability. The type and number of the charges carried by a protein is affected by the pH of its surrounding aqueous environment. These charges affect the electrostatic interactions among the different amino acids in the same protein molecule as well as inter-molecular and molecule-environment interactions. Protein molecules have a neutral net charge at pH values close to their isoelectric points, while they carry positive or negative charges in more acidic or basic conditions, respectively. The stability is unsurprisingly dependent on the balance between attractive/repulsive interactions among the present charges. Exposure of a protein to a pH environment that is far from its isoelectric point, results in strong repulsive forces between its charged groups. Consequently, unfolding may become thermodynamically favorable in this state, as the charge density on the folded protein is higher than on the unfolded protein. In contrast, having a neutral charge reduces the electrostatic repulsion, where hydrophobic attraction between the protein molecules becomes dominant, which could possibly lead to aggregation. Furthermore, specific electrostatic attraction can arise between the charged groups and the oppositely charged ions in the surrounding environment forming salt bridges. This form of interaction has been reported to promote the conformational stability of the protein by stabilizing the folded state in some cases [203].

Of all the BMPs, we were only able to gather pH-dependent stability information for rhBMP-2. The charge distribution of rhBMP-2 is displayed in Fig. 1 [18] and its isoelectric point is 8.2 [23]. Early reports showed loss of rhBMP-2 solubility at pH values above 6 [214, 215], which is probably a part of the reason that the marketed product INFUSE® is formulated at a relatively low pH of 4.5 [216]. A study was conducted to investigate the effect of the formulation pH (4.5 *versus* 6.5) on the conformational stability and aggregation state of rhBMP-2. The analysis was done by using modern complimentary analytical techniques such as intrinsic and extrinsic fluorescence spectroscopy, light scattering, and transmission electron microscopy that look at the 3D structure of the protein as well as its state of aggregation. The results confirmed the loss of solubility at the higher pH as previously reported and indicated the presence of larger size and higher amounts of aggregates accompanied by conformational changes in the higher pH formulation. This was explained by the increased contribution of the hydrophobic attractive interactions by increasing the pH closer to the pI value [23]. The abundance of the surface hydrophobic regions (seen in white in Fig. 1) supports the proposed explanation. It is noteworthy that smaller aggregates (100 nm) were present in the formulation with pH 4.5 as well. This could be an indication of the need for reformulation of this product.

The incorporation of a protein into a delivery system implies changes in the immediate environment of the protein molecules and can alter the type and extent of interactions that may increase or decrease their physical stability. This was demonstrated when the pH of the rhBMP-2 formulation shifted from pH 4.5 to higher pH upon its addition to the collagen sponge [216–218]. Luca *et al.* evaluated the effect of the carrier nature and pH on the *in vivo* osteoinduction of rhBMP-2 in quadriceps muscles of Sprague-Dawley rats. The reconstructed rhBMP-2 solution at pH 4.5 was mixed with either chitosan or HA at two pH values (4.8 and 6.2) for each carrier to form injectable hydrogels. Chitosan and HA are two polymers with similar chemical structures but carrying opposite charges. This means each of them will interact differently when mixed with the positively charged rhBMP-2. Electrostatic attraction would dominate between the negatively charged HA and rhBMP-2, while hydrophobic attraction and hydrogen bonding would govern the interaction between the positively charged chitosan and rhBMP-2. This difference in the interaction types would probably result in different release patterns and possibly different protein stability. rhBMP-2 delivered via both hydrogels was shown to promote bone formation effectively. rhBMP-2 when delivered with HA induced the production of bone with significantly higher level of mineralization, while when delivered with chitosan it resulted in more mature bone. These results indicate that the carrier type indeed has an effect on the quality of the formed bone. Confirming the previously reported pH effect, rhBMP-2 in lower-pH hydrogels (4.8) formed higher mineralized bone compared to the higher-pH hydrogels (6.2) [219], which could be an indication of potential contribution of BMP stability to the observed effect.

In a recent study, a novel composite carrier was developed that was composed of polycaprolactone and type-1 collagen and osteoprogenitor cells and was formulated into a scaffold. However, 2-fold loss in rhBMP-2 bioactivity was reported after mixing with the developed carrier. The addition of heparin and/or bovine serum albumin to rhBMP-2 before its incorporation into the scaffold helped to preserve its bioactivity [220]. This observation is in line with the notion that the choice of carrier type may influence the physical interaction between the protein and carrier material, and thereby the structure and bioactivity of the protein.

There are other causes of protein instability that are only briefly addressed for rhBMPs among the published body of literature. The presence of additives and cosolutes can either physically stabilize or destabilize rhBMPs in aqueous solution according to their type and concentration [203]. The INFUSE® formulation is a lyophilized product containing (after reconstitution) 1.5 mg/ml rhBMP-2 in 5 mM glutamic acid buffer, 2.5% (*w*/*v*) glycine, 5 mM NaCl, 0.5% sucrose, and 0.01% (*w*/*v*) polysorbate 80 [218]. Although formulation developers must have tested other variants and have reasons for choosing this composition, unfortunately, no published information was found about the effects of the additives on protein stability. One study reported that the stability of rhBMP-2 obtained from the marketed product InductOs® (BMP-P) was superior to that from R&D systems (BMP-R) [220]. The BMP-P was reconstituted in sterile water while BMP-R was reconstituted in 4 mM HCl to a final pH of 0. Such extreme acidic conditions likely lead to destruction of the protein prior to any analysis. Shear stress applied to the formulation during transport, reconstruction, and administration (e.g., by injection) has also been mentioned as an important factor to be investigated for its effects on the structural integrity of BMPs [221], although later studies suggested that these effects are likely due to exposure to interfaces rather than shear stress [222, 223].

## Concluding Remarks

Bone morphogenetic proteins present a promising therapy for critical bone defects owing to their excellent capabilities for osteoinduction. However, like other growth factors, their delivery needs to be optimized in terms of the administered dose and their localization at the defect site to improve their efficacy and reduce side effects associated with their pleiotropic actions upon their presence in the systemic circulation. Many of the side effects reported with the use of commercial BMP products (INFUSE® and OP-1) have been connected to their supra-physiological administered dose. Therefore, during the recent years, research has been focusing on the development of carriers with improved release kinetics in order to localize and deliver lower BMP doses.

The side effects reported in clinical trials could well be linked to the properties of the formulation and delivery method as well as the associated instability of BMP, however, these aspects have been mainly overlooked in the published literature. For instance, the current knowledge indicates that the shortage of efficacy and formation of ectopic bone as well as inflammation and prolonged pain can well be related to the non-optimal delivery method and protein instability. Despite the fact that some studies have shown a significant level of success concerning bone formation with lower doses of BMP just by optimization of the formulation and delivery method, the field seldom addresses the potential relation of the carrier type and formulation conditions with preservation of the native structure of the BMP.

Similarly, although the field has been successful in producing several complex composite carriers that were able to clinically induce bone formation, there is a lack of comparative studies differentiating between the types of carriers and their subsequent effect on the formed bone quantity and quality. In fact, the approaches towards the *in vitro* and *in vivo* studies in this area have not significantly changed since the advent of the first BMP product, whereas the understanding of the relation between protein structure and its efficacy and safety as well as formulation effects has been revolutionized in the past decade. Furthermore, enormous progress has been made in invention and employment of novel methods that allow for characterization of proteinaceous growth factors such as BMPs with respect to their primary, secondary, tertiary and quaternary structures as well as their interactions with their environment.

Overall, there is a need for new studies investigating the stability of rhBMPs addressing the current delivery approaches and using complementary analytical techniques to monitor chemical and conformational changes as well as aggregation, e.g., through forced degradation studies [224]. It would be very rewarding to address these points in future studies to understand the relation between formulation and delivery with the protein structure, activity, retention and release and with bone formation quantity and quality. Taking these considerations in future research would provide valuable information that can be used to further enhance the delivery conditions of BMPs, thus enhancing their efficacy and reducing their side effects.

## Abbreviations

3D: Three-dimensional
ACS: Absorbable collagen sponge
ALIF: Anterior lumbar interbody fusion
BMPs: Bone morphogenetic proteins
CMC: Carboxymethylcellulose
ELISA: Enzyme-linked immunosorbent assay
FDA: US Food and Drug Administration
HA: Hyaluronic acid
ICBG: Iliac crest bone grafting
PCL: Poly-ε-caprolactone
PEG: Polyethylene glycol
PGA: Polyglycolic acid
pI: Isoelectric point
PLA: Polylactic acid
PLGA: Poly(D,L-lactide-co-glycolide)
PMA: Premarket approval
PPF: Polypropylene fumarate
TGF-β: Transforming growth factor β
